# Proteins Rpr2 and Pop3 increase the activity and thermal stability of yeast RNase P

**DOI:** 10.1080/15476286.2023.2201110

**Published:** 2023-04-19

**Authors:** Anna Perederina, Igor Berezin, Andrey S. Krasilnikov

**Affiliations:** Department of Biochemistry and Molecular Biology, Center for RNA Biology, Pennsylvania State University, University Park, PA, USA

**Keywords:** Ribonuclease P, RNase P, ribonucleoprotein, RNP, yeast

## Abstract

RNA-based enzyme RNase P is a ribonucleoprotein complex responsible primarily for 5’-maturation of tRNAs. *S. cerevisiae* RNase P comprises a catalytic RNA component and nine proteins. The assembly and maturation of *S. cerevisiae* RNase P involves an abundant and catalytically active precursor form, which includes all components except for proteins Rpr2 and Pop3. Rpr2 and Pop3 are essential proteins, but their roles in RNase P were not clear. Here we use a step-wise in vitro assembly of yeast RNase P to show that the addition of proteins Rpr2 and Pop3 increases the activity and thermal stability of the RNase P complex, similar to the effects previously observed for archaeal RNases P.

## Introduction

RNA-based RNase P [1] is a ubiquitous site-specific endoribonuclease primarily responsible for 5’-maturation of tRNAs. The RNA component of RNase P is the catalytic moiety of the enzyme [2]. The core elements of the RNA component responsible for the pre-tRNA substrate recognition and cleavage are structurally conserved in all domains of life, whereas the protein complement differs significantly [3–5]. The *S. cerevisiae* RNase P holoenzyme includes RPR1 RNA (369 nucleotides-long) and 9 protein components: Pop1 (100.5 kDa), Pop3 (22.6 kDa), Pop4 (32.9 kDa), Pop5 (19.6 kDa), Pop6 (18.2 kDa), Pop7 (15.8 kDa), Pop8 (15.5 kDa), Rpp1 (32.2 kDa, 2 copies), and Rpr2 (16.3 kDa). Yeast RNase P shares all of its proteins except for Rpr2 with its sister enzyme, RNase MRP [6]. All RNase P proteins are essential in yeast [6].

A partial stepwise reconstruction of yeast RNase P RNP including all components except for proteins Rpr2 and Pop3 has been previously described [7]. This reconstruction, combined with the cryo-EM structure of the enzyme [8], has allowed the characterization of the structural and functional roles of most of the yeast RNase P proteins. The largest yeast RNase P protein Pop1 serves as the major structural brace stabilizing the global fold of RNase P RNA and is required for the catalytic activity of RNA *in vitro* [7–9]. Proteins Pop6 and Pop7 form a heterodimer that mediates additional interactions between Pop1 and RNA [7–10]. Protein Pop4 bridges the catalytic and specificity domains of RNase P RNA [8] and dramatically increases the activity of the RNP *in vitro* [7]. Proteins Pop5, Pop8, and 2 copies of Rpp1 form a heterotetramer that extensively interacts with RNase P RNA and contributes to the formation of the substrate-binding pocket; Pop5 and Rpp1 are required for the RNP activity, while the presence of Pop8 considerably increases it [7,8]. Rpr2 and Pop3 bind at the periphery of the complex; they were absent in the RNase P reconstruction experiments [7] and their effect on the activity of the yeast enzyme was unknown.

**Figure 1.**
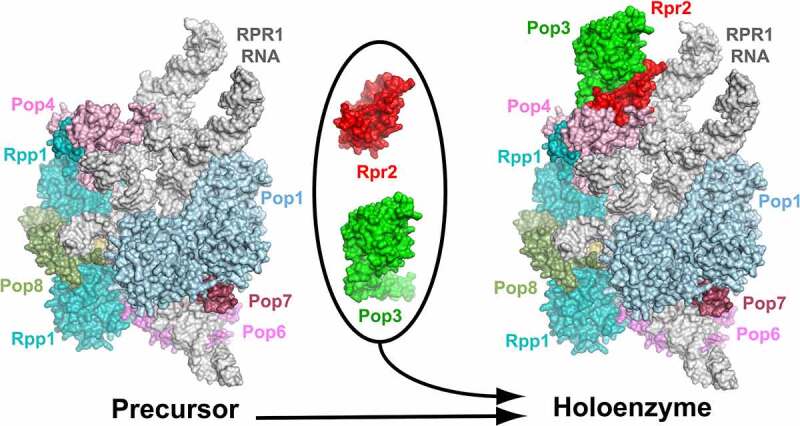
Formation of the mature form of yeast RNase P from the precursor molecule involves the binding of proteins Rpr2 and Pop3. RNA is shown in grey. Figure is based on 6ah3.pdb [8].

The assembly and maturation of yeast RNase P involves a precursor that is missing peripheral protein components Rpr2 and Pop3 [11] (Figure 1). The precursor form constitutes 10–20% of all cellular RNase P and is catalytically active [11]. The roles of yeast Rpr2 and Pop3 (which are essential for the viability of the cell [6]) were not clear. Here we used *in vitro* reconstruction of RNase P to investigate the effects of the addition of Rpr2 and Pop3 on the activity and thermal stability of *S. cerevisiae* RNase P.

## Materials and methods

**Figure 2.**
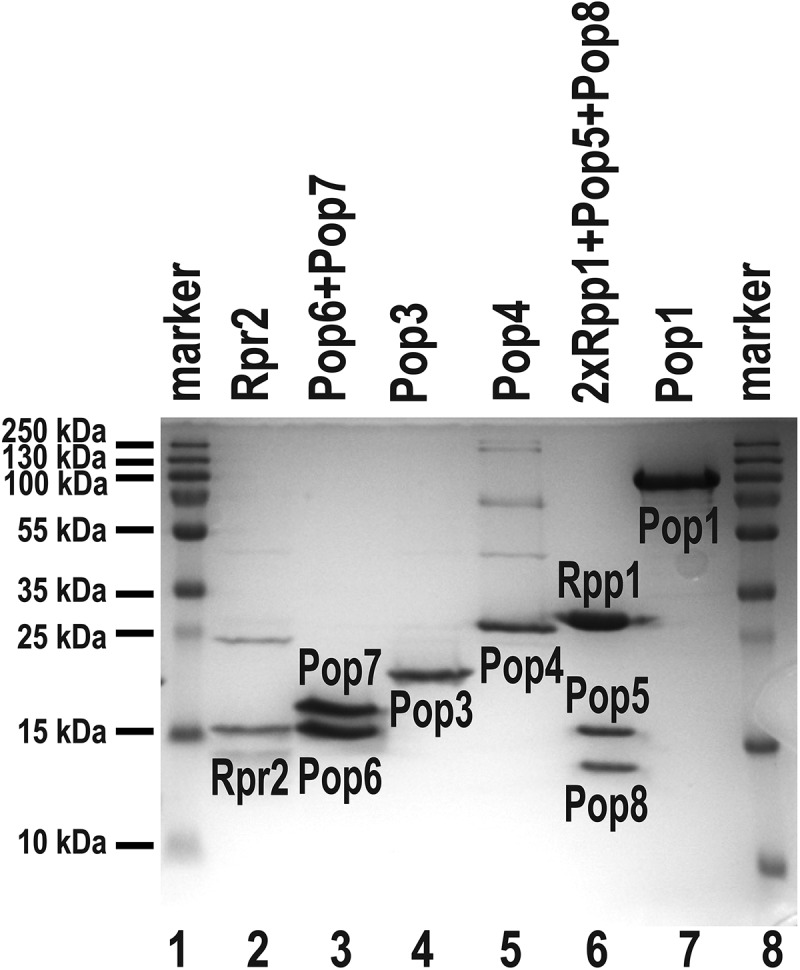
Isolation of protein components of yeast RNase P. Protein Pop1 was co-expressed with Pop4, but purified separately [7,9]; proteins Rpr2, Pop3, and Pop4 were expressed and purified individually [7]; complexes Pop6/Pop7 and 2×Rpp1/Pop5/Pop8 were obtained using co-expression of corresponding protein components [7,10]. Lanes 1, 8: size marker; lane 2: Rpr2; lane 3: Pop 6/Pop7 complex; lane 4: Pop3; lane 5: Pop4; lane 6: 2×Rpp1/Pop5/Pop8 complex; lane 7: Pop1. Coomassie Blue-stained SDS-polyacrylamide gel.

The components of RNase P were produced as previously described [7]. All proteins were overexpressed in *Escherichia coli* (Figure 2).

Proteins Pop4, Pop3, Rpr2 were fused to the C-terminus of maltose-binding protein (MBP) via a linker containing a tobacco etch virus (TEV) protease cleavage site. The MBP tag was used for the purification of these proteins and removed using TEV protease.

Proteins Rpp1, Pop5, Pop8 were co-expressed from a single polycistronic construct and purified as a 2×Rpp1, Pop5, Pop8 tetramer [7].

Proteins Pop6, Pop7 were co-expressed and purified as a heterodimer [10].

To obtain protein Pop1, it was co-expressed with Pop4. Co-expression of the two proteins was required to obtain soluble Pop1; neither of the co-expressed proteins had any purification tags. The co-expressed Pop4 was separated from Pop1 in the process of Pop1 purification and discarded [9].

Final isolated proteins did not include any purification tags; however, Pop4, Pop3, and Rpr2 had an additional glycine residue at their respective N-terminus due to the used purification scheme.

The RNA component of RNase P was produced using run-off *in vitro* transcription with a linearized plasmid pYRP2 [7] as the template. pYRP2 carries a deletion of non-conserved nucleotides A24, U25 to facilitate proper RNA folding; the deletion did not affect the assembly and activity of the RNP [7].

The assembly of the RNase P RNP complexes was performed in a stepwise fashion by the addition of proteins to an equimolar amount of refolded RNase P RNA as previously described [7]. Concentrations of components were determined spectrophotometrically using NanoDrop ND-1000 spectrophotometer (Thermo Scientific). RNA concentration was calculated based on the absorbance at 260 nm and using the absorbance coefficient 0.025 (μg/ml)^−1^/cm. Concentrations of proteins were calculated based on absorbance at 280 nm; the absorbance coefficients were calculated using ExPASy ProtParam tool (http://www.expasy.org [7,12]). Proteins were added sequentially as follows: Pop6/Pop7, Pop1, Rpp1/Pop5/Pop8, Pop4, Rpr2, Pop3. Attempts to pre-incubate complexes with a substrate or at a higher temperature [7] did not improve the result.

*In vitro* transcribed *S. cerevisiae* pre-tRNA^Thr^(AGT) construct [7] was used as the substrate in the cleavage assays. This pre-tRNA included a 15-nucleotide 5’-leader and a 10 nucleotide-long 3’-trailer; all sequences corresponded to the *S. cerevisiae* genome (chromosomal location chrIII:295469–295565, http://www.yeastgenome.org/cgi-bin/seqTools). In addition, a 5’-end GG was added to facilitate transcription [7]. Prior to the use in cleavage assays, the substrate was annealed by heating to 50°C followed by slow cooling to 25°C.

Cleavage assays were performed in a buffer containing 50 mM HEPES-NaOH pH 7.8, 100 mM ammonium acetate, 10 mM MgCl_2_, 0.1 mM EDTA, 1 mM DTT, 0.5% (v/v) glycerol, 0.5 μg/ml BSA. An aliquot of the pre-tRNA substrate (above) was 5’-^32^P-labelled, mixed with the cold substrate, and added to the reaction mix to the final substrate concentration of 4 μM. The reactions were run at varying temperatures for varying time with RNase P RNPs of varying compositions and concentrations, and stopped by the addition of an equal volume of a stop/loading buffer containing 8 M urea and 25 mM Na-EDTA pH 8.0. The products of the reactions were resolved on 8% denaturing polyacrylamide gels and quantified using a PhosporImager (Molecular Dynamics). The assays were run in triplicates at 30°C and 45°C, and in duplicated at all other temperatures.

Control RNase P holoenzyme was isolated from *S. cerevisiae* using a tandem affinity tag fused to the C-terminus of protein Rpr2 as previously described [7].

## Results and discussion

We have assembled an RNP complex consisting of RNase P RNA and proteins Pop1, Pop4, Pop5, Pop6, Pop7, Pop8, and Rpp1 (2 copies) (termed RNP_7_ here) using our previously described approach [7]. The protein composition of RNP_7_ matches that of the abundant precursor of yeast RNase P, while the mature enzyme contains additional proteins: Rpr2 and Pop3 [11]. To elucidate the roles of Rpr2 and Pop3, we have added these proteins to the RNP_7_ complex and investigated their effect on the activity and thermal stability of the enzyme.

**Figure 3.**
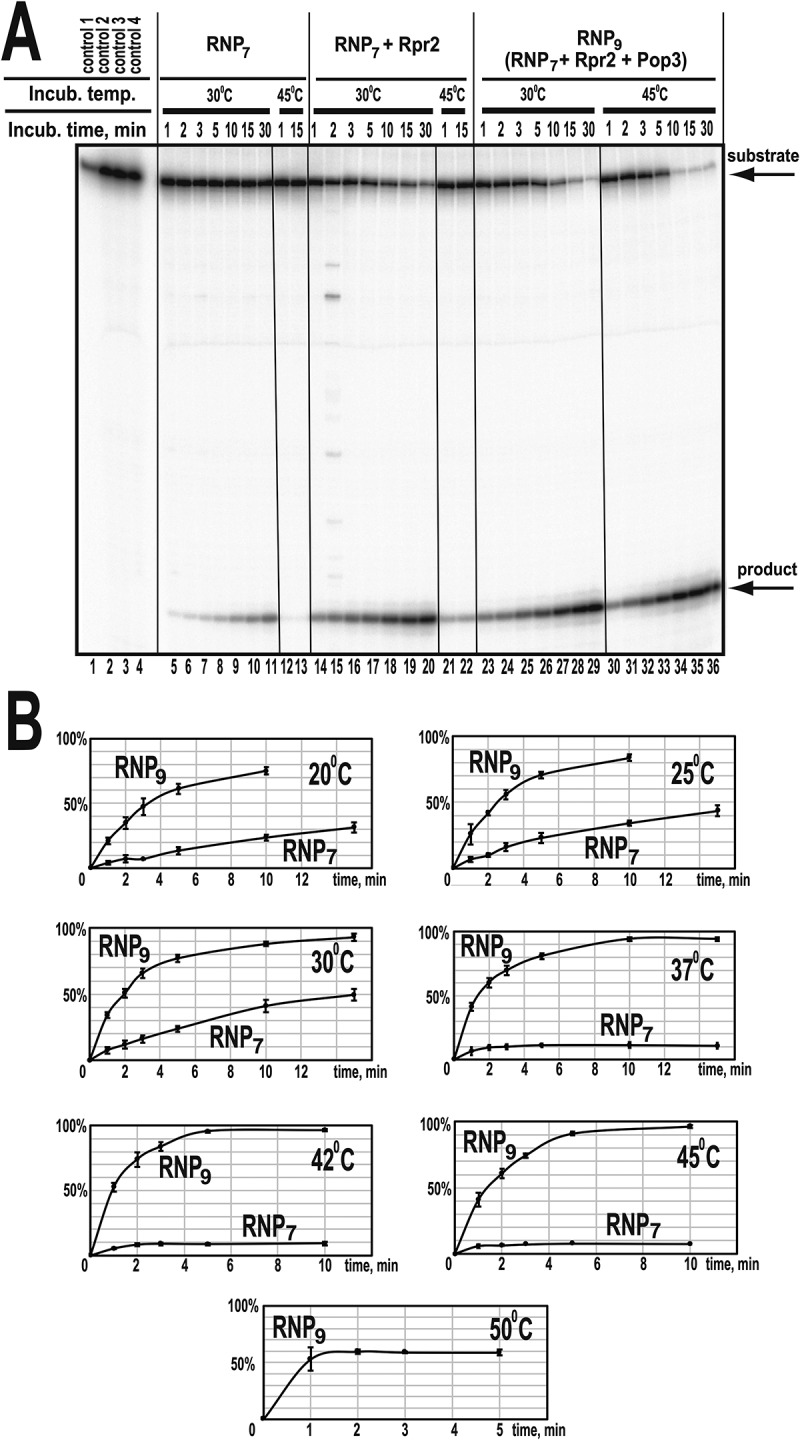
(A) Time course of the cleavage of pre-tRNA substrate by reconstructed RNase P RNP complexes RNP_7_ (RNase P RNA, Pop1, Pop4, Pop5, Pop6, Pop7, Pop8, Rpp1) (lanes 5–13), RNP_7_ plus protein Rpr2 (lanes 14–22), and RNP_9_ (RNP_7_ with added Rpr2 and Pop3) (lanes 23–36). pre-tRNA^Thr^ substrate (at 4 μM) was cleaved by RNPs (at 40 nM) at the temperatures indicated above the corresponding lanes. Lanes 1–4: negative controls, incubation at 30°C for 15 min. Lane 1: substrate; lane 2: substrate in the cleavage buffer; lane 3: substrate with RNase P RNA; lane 4: substrate with all proteins. (B) Quantification of pre-tRNA cleavage (gels are shown in Appendix Fig. S1). pre-tRNA^Thr^ substrate (at 4 μM) was cleaved by of RNP_7_ and RNP_9_ (at 100 nM) at the indicated temperatures. Note that the cleavage by RNP_7_ at 37–45°C and by RNP_9_ at 50°C occurs only in the first two minutes of the incubation while the sample tube is warming to the set temperature and does not proceed further apparently due to the thermal inactivation of the enzyme.

Consistent with the previously reported results [7], the RNP_7_ complex is catalytically active (Figure 3 and Appendix Fig. S1A). This observation is also consistent with the previously reported catalytic activity of the precursor form of RNase P that misses Rpr2 and Pop3 [11].

Interestingly, activity assays (Figure 3 and Appendix Fig. S1A) show that while RNP_7_ is active at temperatures up to 30°C, the activity is lost at 37°C, indicating a reduced thermal stability of the complex with the protein composition matching that of the RNase P precursor. It was observed that incubation of RNP_7_ at 37°C and above resulted in visible precipitation of the complex. (It should be noted that in experiments under impermissible temperatures, about 10% of the substrate is cleaved in the first two minutes while the sample tube is warming to the set temperature, but the cleavage does not proceed further, Figure 3b.)

Whereas the assembly of RNP_7_ resulted in a quantitative binding of the components with the formation of a structurally homogeneous complex, the addition of Rpr2 and Pop3 did not result in a quantitative binding as judged by gel mobility shift and gel filtration experiments, suggesting that a stable binding of these proteins may require additional chaperoning activity [7]. Nevertheless, here we detected a substantial effect of the addition of Rpr2 and Pop3 on the thermal stability and the observed activity of the RNase P RNP.

The addition of Rpr2 and Pop3 to RNP_7_ resulted in a considerable increase of the thermal stability of the complex: robust activity is observed at temperatures up to 45°C; the complex is inactivated by 50°C (Figure 3b, Appendix Fig. S1C). Incubation of the complex at 50°C resulted in a noticeable precipitation, indicative of thermal denaturation. Interestingly, this level of thermal stability appears to be somewhat excessive for a *S. cerevisiae* enzyme, given that *S. cerevisiae* strains typically do not grow at temperatures above 40°C [13].

Also, the addition of proteins Rpr2 and Pop3 resulted in a considerable increase of the activity of the complex. Compared to the fully assembled RNase P isolated from yeast, the RNP_7_ complex had a reduced level of activity: *k*_cat_ = 2.0 ± 1.0 min^−1^, *K*_m_ = 3.5 ± 1.5 μM *vs k*_cat_ = 40 ± 20 min^−1^, *K*_m_ = 50 ± 20 nM (estimated at 30°C) [7]. The non-quantitative character of the binding of Rpr2 and Pop3 does not allow for a meaningful detailed estimation of the kinetic parameters of the substrate cleavage in the presence of these proteins; however, the observed rate of cleavage at 20°-30°C and the concentration of the substrate of 4 μM was at least 5-fold higher than that for the RNP_7_ complex (Figure 3, Appendix Fig. S1). The remaining difference between the rates of pre-tRNA cleavage by the isolated yeast RNase P versus the assembled complexes is consistent with the non-quantitative binding of Rpr2 and Pop3. Nevertheless, the substantial increase of the cleavage rate even with the incomplete Rpr2 and Pop3 binding indicates the role of these proteins.

**Figure 4.**
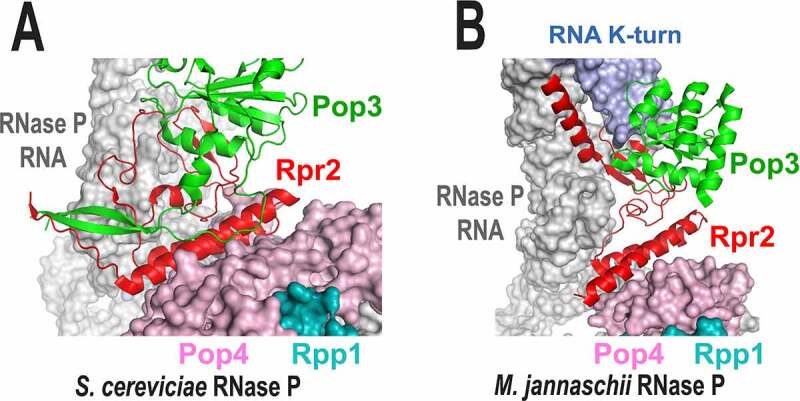
In both yeast (panel A) and archaeal (panel B) RNases P, protein Rpr2 (red) is sandwiched between Pop3 (green) and the rest of the RNP and is involved in extensive interactions with the RNA component (grey) and protein Pop4 (magenta). Archaeal protein Pop3 is involved in interactions with the K-turn RNA motif (blue in panel B) that is absent in the yeast RNase P. Panel a is based on 6ah3.pdb [8]. Panel B is based on 6k0a.pdb; the structure of Methanocaldococcus jannaschii RNase P is shown [17]. Shown names of archaeal proteins correspond to the names of their yeast homologues.

The observed increase of the activity and thermal stability of yeast RNase P in the presence of Rpr2 and Pop3 is consistent with the available structural data and parallels the effects observed for archaeal RNase P [14–18]. Both in yeast and archaeal enzymes, Rpr2 is sandwiched between Pop3 and the rest of the molecule (Figures 1, 4), forming extensive interactions with the part of RNase P RNA that is involved in the pre-tRNA substrate binding and is positioned to stabilize the fold of that part of RNA and to bridge it to the rest of the molecule. Additionally, Rpr2 interacts with the T-loop region of the pre-tRNA substrate, contributing to the proper positioning of the substrate. These roles can explain the effect of Rpr2 on the activity of RNase P.

The structure of Rpr2 in the context of yeast RNase P (Figure 4a) suggests the instability of the protein’s fold in the absence of Pop3, which is consistent with the observed propensity of isolated Rpr2 to aggregate. Pop3 in involved in extensive interactions with Rpr2, including the insertion of antiparallel N- and C-terminal beta-strands into the fold of Rpr2 (Figure 4a) and is positioned to stabilize it. This stabilization is consistent with the role of Pop3 in the thermal stability of yeast RNase P.

Given that yeast Pop3 does not participate in extensive interactions with components of RNase P other than Rpr2 [17], its role in the yeast enzyme is likely limited to the stabilization of Rpr2 and facilitation of its binding to the complex. Consistent with that, we did not observe any effects of Pop3 on the activity of the complex in the absence of Rpr2. It should be noted that in archaeal RNase P, the addition of the constitutive homologue of Pop3 (L7Ae) increases the activity and thermal stability of the enzyme as well [14,15]. In archaeal RNase P, Pop3 (L7Ae) contributes to the stability of the enzyme through its interactions with K-turn motif(s) of the RNA component as well as through extensive interactions with the homologue of Rpr2 [17–19] (Figure 4b). Yeast RNase P has lost the K-turn RNA motifs (Figure 4), thus minimizing interactions between Pop3 and RNA, but it has retained Pop3-Rpr2 interactions and the overall role of Pop3 in the stability of the enzyme.

## Supplementary Material

Supplemental Material
