# Supplementary material for: Proteins Rpr2 and Pop3 increase the activity and thermal stability of yeast RNase P
Source: RNA Biol. 2023 Apr 19;20(1):149–53. doi: 10.1080/15476286.2023.2201110 (PMC10120536; doi:10.1080/15476286.2023.2201110)
Supplement: Supplemental Material [file KRNB_A_2201110_SM8918.docx]

**Proteins Rpr2 and Pop3 increase the activity and thermal stability of yeast RNase P**

Anna Perederina, Igor Berezin, Andrey S. Krasilnikov

*Department of Biochemistry and Molecular Biology*, *Center for RNA Biology*,

*Pennsylvania State University*, *University Park, PA 16802, USA*

**Appendix**

**Appendix Figure S1.** Time course of the cleavage of pre-tRNA substrate by reconstructed RNase P RNP complexes RNP_7_ (RNase P RNA, Pop1, Pop4, Pop5, Pop6, Pop7, Pop8, Rpp1) (panel A) and RNP_9_ (same as RNP_7_ plus Rpr2 and Pop3) (panel C). Lanes 1-34 and 37-74: pre-tRNA substrate (at 4 μM) was cleaved by RNPs (at 100 nM) at the temperatures indicated above the corresponding lanes. Lanes 35, 36 (panel B): cleavage by RNP_9_ and the RNase P holoenzyme isolated from yeast (control). Quantification of the cleavage is presented in Fig. 3B of the main text.
